# Empirical prediction intervals applied to short term mortality forecasts and excess deaths

**DOI:** 10.1186/s12963-024-00355-9

**Published:** 2024-12-11

**Authors:** Ricarda Duerst, Jonas Schöley

**Affiliations:** 1 Max Planck Institute for Demographic Research, Konrad-Zuse-Straße 1, 18057 Rostock, Germany; 2 University of Helsinki, Fabianinkatu 33, 00014 Helsinki, Finland

**Keywords:** Excess deaths, COVID-19, Cross-validation, Robustness, Empirical prediction intervals

## Abstract

**Background:**

In the winter of 2022/2023, excess death estimates for Germany indicated a 10% elevation, which has led to questions about the significance of this increase in mortality. Given the inherent errors in demographic forecasting, the reliability of estimating a 10% deviation is questionable. This research addresses this issue by analyzing the error distribution in forecasts of weekly deaths. By deriving empirical prediction intervals, we provide a more accurate probabilistic study of weekly expected and excess deaths compared to the use of conventional parametric intervals.

**Methods:**

Using weekly death data from the Short-term Mortality Database (STMF) for 23 countries, we propose empirical prediction intervals based on the distribution of past out-of-sample forecasting errors for the study of weekly expected and excess deaths. Instead of relying on the suitability of parametric assumptions or the magnitude of errors over the fitting period, empirical prediction intervals reflect the intuitive notion that a forecast is only as precise as similar forecasts in the past turned out to be. We compare the probabilistic calibration of empirical skew-normal prediction intervals with conventional parametric prediction intervals from a negative-binomial GAM in an out-of-sample setting. Further, we use the empirical prediction intervals to quantify the probability of detecting 10% excess deaths in a given week, given pre-pandemic mortality trends.

**Results:**

The cross-country analysis shows that the empirical skew-normal prediction intervals are overall better calibrated than the conventional parametric prediction intervals. Further, the choice of prediction interval significantly affects the severity of an excess death estimate. The empirical prediction intervals reveal that the likelihood of exceeding a 10% threshold of excess deaths varies by season. Across the 23 countries studied, finding at least 10% weekly excess deaths in a single week during summer or winter is not very unusual under non-pandemic conditions. These results contrast sharply with those derived using a standard negative-binomial GAM.

**Conclusion:**

Our results highlight the importance of well-calibrated prediction intervals that account for the naturally occurring seasonal uncertainty in mortality forecasting. Empirical prediction intervals provide a better performing solution for estimating forecast uncertainty in the analyses of excess deaths compared to conventional parametric intervals.

## Introduction

In the winter of 2022/2023, excess death estimates for Germany were hovering around 10%. Back then, the authors have been contacted by journalists inquiring about the significance of the elevated mortality. Given that statistical estimation always comes with errors attached, can one even reliably estimate a 10% deviation from the norm? In this paper we aim to answer this question via the careful analysis of the error distribution in forecasts of weekly deaths and the seasonality of fluctuations in weekly death counts. Derived from the distribution of errors we propose empirical prediction intervals for the probabilistic study of weekly expected and excess deaths and demonstrate the superior coverage and generality of these intervals compared with conventional parametric intervals. We employ these empirical prediction intervals to quantify, for a range of countries, the probability of observing at least $10\%$ excess deaths in a given week given the continuation of mortality trends observed prior to the COVID-19 pandemic. Using these *p*-values we can assess, on a per-country basis, how unusual a 10% mortality increase over the expectation is and whether it should be cause for concern.

Mortality forecasts on a sub-annual timescale have gained relevance as the basis for excess death calculations during the COVID-19 pandemic (e.g. [1–3]). Framed as a forecasting problem, one aims to predict the weekly deaths which would have happened without COVID-19 by forecasting deaths over the pandemic period based on pre-pandemic trends. Those forecast ”expected deaths“ are associated with an error which can be expressed as a ”prediction interval“ within which the true expected deaths are to be found with a given probability. It is well known that prediction intervals around forecast values tend to be too narrow (e.g. [4, 5]). In the context of COVID-19 excess death modeling, this phenomenon may lead to wrong conclusions regarding the impact of the pandemic on population mortality, by giving an overly optimistic picture on how precise one can actually forecast counterfactual weekly expected deaths more than three years since the onset of COVID-19. Precisely, given the time passed since the beginning of the pandemic, we expect the uncertainty of forecast expected deaths to have increased. Thus, one might (or should) ask whether we can still, in fall of 2022, reliably detect an e.g. 10% increase in excess deaths or whether it disappears into the uncertainty of the expected deaths forecasts.

To answer this question, we need reliable measures of forecast uncertainty. It is common practice in demographic forecasting to use parametric prediction intervals, derived from the model structure, such as a random walk over the mortality index of a Lee-Carter model [6], or the Poisson/negative-binomial variation around weekly death counts [3, 7]. However, of the various sources of error that can contribute to the overall forecast uncertainty (see [8] for an overview), these parametric prediction intervals do not reflect errors from the out-of-sample generalization of the model or inaccurate model specification. This is particularly problematic as the out-of-sample generalization error is the most common type of forecast error [8]. Therefore, the parametric intervals only work if the model is correctly specified and the data generation process does not change over time. As a result, parametric intervals tend to be too narrow.

Empirical prediction intervals, instead of relying on correct parametric specifications, estimate the distribution of error around a forecast value from actual past, out-of-sample forecasting errors. The idea is simple: The error distribution for future forecasts will be similar to the error distribution of past forecasts. Thus, one seeks to estimate the full distribution of forecast error indexed over all desired forecasting strata and time points. In order to do so, a statistical model is fitted to known out-of-sample forecasting errors. This error distribution can then be used to construct empirical prediction intervals around the central forecast.

Research on empirical prediction intervals has been ongoing for at least half a century [9]. The 1980s saw active demographic research on empirical intervals and the authors generally found that the intervals based on past errors had better coverage than model-based intervals (e.g. [10–12]). Subsequent research used the analysis of forecasting errors to calibrate prediction intervals for demographic forecasts. A summary of methodological advances (e.g. [8, 13, 14]) and demonstrations of the application of the method can also be found in more recent demographic literature (e.g. [15–18]).

In the machine learning literature, there is growing interest in ”distribution free uncertainty quantification“, given that popular techniques do not provide an intrinsic estimate of uncertainty. The community has build a theory on empirical prediction intervals under the term ”conformal prediction“. Here too, the idea is to look at the distribution of historical error measures to assess how likely any given future deviation from the central prediction would be [19].

In this paper, we demonstrate the construction of empirical prediction intervals for short-term mortality forecasts. We validate these intervals against parametric approaches and interpret the results in the context of COVID-19 excess death estimation across 23 European countries. The data on weekly deaths for these countries are sourced from the Short-term Mortality Fluctuations Database (STMF, [20]). Finally, we determine whether we can reliably detect a 10% increase in excess deaths in the fall of 2022, given the empirical uncertainty around the expected deaths forecasts.

## Data and methods

### Data

We sourced data on weekly deaths from the Short-Term Mortality Fluctuations Database (STMF) [21]. The STMF is part of the Human Mortality Database (HMD) [22] and was established in response to the COVID-19 pandemic and the “increasing importance of short-term or seasonal mortality fluctuations that are driven by temporary hazards such as influenza epidemics, temperature extremes, as well as man-made or natural disasters” [20]. The STMF provides open-access data on mortality by week, sex, and aggregated age group for 38 countries that follow the HMD’s criteria of high data quality. Information on the data quality, sources, and completeness for each individual country can be found in the metadata file [23]. In general, STMF data for all countries is characterized by high quality, timely registration and completeness. The mortality counts are neither adjusted for e.g., under-reporting, nor are they smoothed [20]. We use weekly death counts for 23 of the available countries. Because the data availability across time varies by country, we selected countries whose first observed data entry is for the year 2000 or before. These long time series are needed to robustly estimate the empirical error distribution, because the data is cut into several cross-validation series, which will be described in detail in this section.

**Fig. 1:**
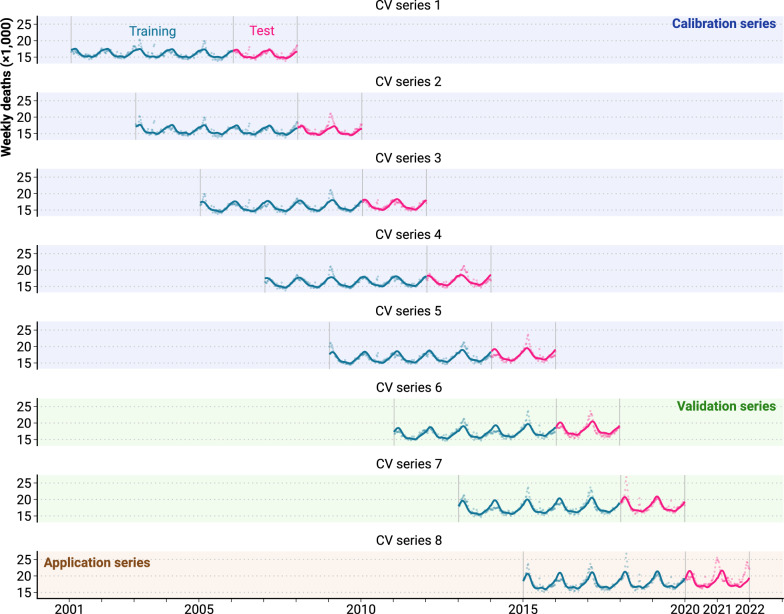
Data-splitting setup. 22 years of weekly death counts have been split into 8 overlapping cross-validation data series (CV series). Each series features 5 years of training data and 2 years of test data. The empirical forecasting error is estimated from the test set of the calibration series, validated on the test set of the validation series and applied to the test set of the application series

Split the data for each country into three different types of data series (see Fig. 1). We use these data series for different parts of our analysis. The first five data series are the calibration series. Using these, we calibrate the empirical prediction intervals. The following two series are used for the validation of the derived empirical prediction intervals. The forecast period of the last data series spans over the time of the COVID-19 pandemic and is the application series. Using the application series, we answer our research question on detecting an increase in excess deaths given the uncertainty of the forecasts of expected deaths.

Starting from the last observed week, we split the data into eight partially overlapping series of 365 weeks (7 years). Each series is divided into a training period (blue) of 261 weeks (5 years), and a forecast period (pink) of 104 weeks (2 years) (see Fig. 1 for Germany and Table 2 for the starting and end dates of the data series).

### Steps of analysis

The methodology of our analysis is structured into several steps. With the first four steps, we obtain and calibrate the empirical prediction intervals, and parametric prediction intervals for comparison. For this, we use the calibration data series. During step five, we assess the calibration of the prediction intervals using the validation data series. Following these steps, we are able to answer our research question regarding the application of empirical prediction intervals to the COVID-19 pandemic.

**I Modeling and forecasting expected deaths**

First, we model and forecast the expected deaths. These are the deaths that would have occurred if a specific event e.g., the COVID-19 pandemic, did not happen. In line with the WHO approach [24], we forecast expected deaths $\hat{y}^{E}$ at time $T+h$ using an overdispersed count regression,

1 $$\begin{aligned} \hat{y}^{E}_{T+h} = \textrm{exp}\left( \alpha +\beta _{h}h+s_{w}\left( w\left[ h \right] \right) \right) , \end{aligned}$$

where *T* is the last time-point in the training data and *h* is the number of weeks into the forecasting horizon, $\beta _{h}h$ captures the long term trend of deaths, and $s_{w}\left( w\left[ h \right] \right)$ is a cyclical penalized spline over the week of the year to reflect the seasonality of death counts. The model is fitted to the training periods of the data series and predictions are made over the testing periods. Note that empirical prediction intervals can be derived for any other model for excess deaths. For simplicity of exposition we did not include further refinements like population offsets, temperature effects, or auto-correlated errors into the model. We assume the weekly deaths under the expected scenario to be distributed as

2 $$\begin{aligned} Y_{T+h}^E \sim \mathrm {Neg.Binomial}(\hat{y}^{E}_{T+h}, \phi ). \end{aligned}$$

**II Deriving the forecast error**

In a second step, we quantify the forecasting error at time $T+h$ as the logged ratio between observed and expected deaths over the test period:

3 $$\begin{aligned} u_{T+h} = \log \frac{y^O_{T+h}}{\hat{y}^E_{T+h}}. \end{aligned}$$

We choose the log-ratio as the error scoring function because it reduces the scale dependence and asymmetry of the distribution of errors, in turn making it easier to model the observed error distribution.

**III Modeling the forecast error**

Third, we model the time-varying distribution of the observed forecasting error over the forecasting horizon, $U_{T+h}$, via a skew-normal distribution with a location parameter $\mu$, and time-varying scaling and skewness parameters $\sigma _h$ and $\upsilon _h$:

4 $$\begin{aligned} U_{T+h} \sim \textrm{SkewNormal}(\mu , \sigma _h, \upsilon _h). \end{aligned}$$

As the observed log-errors are positively skewed, the skew-normal distribution allows for more accurate modeling of the error distribution over time, compared to a normal distribution.

We model the scaling parameter as

5 $$\begin{aligned} \sigma _{h} = \exp \left( \alpha _{\sigma }  + s_\sigma (w[h]) \right) \end{aligned}$$

where $s_\sigma (w[h])$ is a smooth function of calendar week, capturing the annual seasonality of the error dispersion. In the context of expected deaths modeling, this seasonality is pronounced due to the challenges in predicting the severity of flu-waves during winter. While we would expect the error variance to increase with increasing forecasting horizon as well, we found that effect to be negligible over the duration of 100 weeks and did not include it here. The skewness $\upsilon _{h}$ is modeled equivalently via

6 $$\begin{aligned} \upsilon _{h} = \alpha _{\upsilon } + s_\upsilon (w[h]), \end{aligned}$$

and the mean error is captured in the constant

7 $$\begin{aligned} \mu = \beta _{\mu _0}. \end{aligned}$$

**IV Deriving prediction intervals**

Once estimates for $\sigma _h$, $\mu _h$ and $\upsilon _h$ are found, in the fourth step, we derive the distribution of expected deaths from the quantiles of the error distribution. The *p* quantile of the distribution of expected/forecasted deaths $Y^E_{T+h}$ can be derived from the corresponding quantile of the error distribution via

8 $$\begin{aligned} Q_{Y^E_{T+h}}(p)=\exp (\hat{F}_{U_{T+h}}^{-1}(p))\cdot \hat{y}^E_{T+h}, \end{aligned}$$

where $\hat{F}_{U_{T+h}}^{-1}$ is the estimated distribution function of the forecasting error. Intuitively, if we estimate that 95% of the errors in our forecast for a given week do not exceed 1.5 times the central forecast, then the corresponding 95% prediction interval around the expected deaths is 1.5 times the central forecast for that week, likewise for other quantiles.

For comparative validation, we also derive prediction intervals from a negative-binomial generalized additive model (GAM). The negative-binomial GAM prediction intervals are commonly used for count data (see [25] for examples). Additionally, we calculate the raw quantiles of the forecast error distribution as a second empirical model of the forecast error.

**V Assessing calibration of empirical prediction intervals**

In the final step, we assess the calibration of the empirical and parametric prediction intervals using two different calibration metrics: the coverage, and the mean interval score.

The coverage measures the fraction of observations that are within the bounds of the prediction interval. Ideally, nominal coverage and observed coverage are the same, i.e. a 95% prediction interval should, over the course of many forecasts, cover 95% of realized values. For a collection of prediction intervals $(l_i, u_i)$ and associated observations $y_i$ the coverage can be calculated as

9 $$\begin{aligned} \textrm{Coverage} = \frac{\sum _i^N\textbf{1}(l_i< y_i < u_i)}{N} \end{aligned}$$

where $l_i$ and $u_i$ are the $\frac{\alpha }{2}$ and $1-\frac{\alpha }{2}$ quantiles, $i=1,\ldots , N$ is an index over all forecast data points, and $\textbf{1}(\cdot )$ is the indicator function.

The mean interval score as proposed by Gneiting and Raftery [26] is an example of a *proper scoring rule* and as such has optimal properties for assessing the quality of distributional and, specifically, interval forecasts. In the literature on COVID related prediction models the interval score has seen intensive use [27–30], and is one of the evaluation measures of the COVID-19 Forecasting Hub Project [31].

The mean interval score balances the two requirements of coverage and sharpness, rewarding narrower prediction intervals but penalizing deviations from the nominal coverage. Among two alternative prediction intervals with equal coverage, the interval which on average is narrower will yield the lower, i.e. better, mean interval score. This balancing property is very valuable in the evaluation of prediction intervals around forecasts of weekly deaths, because the variance of the error varies with the season. Thus, it is possible to have prediction intervals which have perfect coverage over the whole year but are too narrow in winter and too wide in spring. The mean interval score instead rewards prediction intervals that are narrow where they can be, and wide where they must be. The score is defined as follows:

10 $$\begin{aligned} S_{\alpha ,i}(l_i, u_i, y_i) = \left\{ \begin{array}{rcl} (u_i-l_i)+\frac{2}{\alpha }(l_i-y_i) & \text{ for } & y_i<l_i \\ (u_i-l_i)+\frac{2}{\alpha }(y_i-u_i) & \text{ for } & y_i>u_i \end{array}. \right. \end{aligned}$$

We then average the interval scores over all forecasts in the validation series across all countries. Following a suggestion by Bosse et al. [32], we calculate the interval scores over log-transformed observations and prediction intervals, as otherwise the mean interval score would be dominated by countries with large population numbers.

## Results

### Method demonstration for Germany

In the following, we will showcase the application of the empirical prediction interval methodology for Germany. The results of the cross-country analyses will be presented in the following sections.

**Fig. 2:**
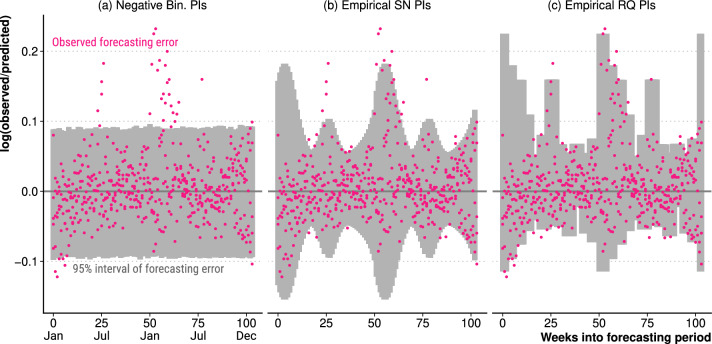
Distribution of the observed forecast errors in the calibration data series (pink, test periods from 2006 to 2016), with modelled forecast error distributions, for Germany

Following steps I to III of our methodology, Fig. 2 shows the distribution of the weekly observed forecast errors in the data series used for calibration, along with modelled forecast error distributions resulting from three different models. Panel (a) shows the observed forecast errors contrasted against the 95% error interval derived from the negative-binomial variation, a common parametric specification in excess death modelling [3, 7]. Panels (b) and (c) show two types of post-hoc (empirical) estimates of the forecast error distribution: the skew-normal error model (b) and the raw quantiles of the empirical error distribution (c).

As expected, the observed errors (pink) are positively skewed with a strong seasonal pattern. Both the skewness and the seasonality in the distribution of forecasting errors, with elevated errors in winter (weeks 0–12, 49–66 and 95–104) and, to a lesser extent, in summer (weeks 20–30 and 75–85), are due to the irregularities in the annual occurrence of flu-epidemics and heat-waves. This seasonal shift in the variance of forecasting error is well reflected by the skew-normal model (b) and the empirical quantiles (c). In contrast, the often used negative-binomial specification with fixed overdispersion implies a constant forecasting error (a). Figure 5 in the Appendix shows the forecast error distribution modelled with the skew-normal distribution for all 23 countries.

**Fig. 3:**
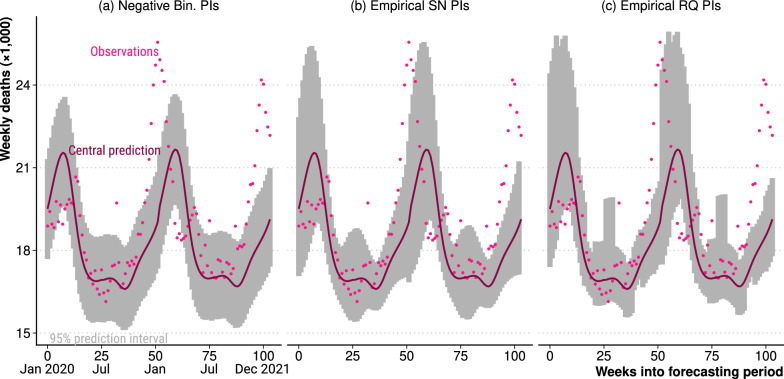
Negative-binomial prediction intervals (**a**), and empirical prediction intervals (**b, c**) applied to the application (COVID-19) data series (test periods from 2020 to 2021), for Germany

As specified in step IV, we derive empirical prediction intervals around the central forecasts of expected deaths from the estimated distribution of forecasting error. For the negative-binomial model, we simply report the parametric prediction intervals. Figure 3 contrasts the three approaches in the context of the German COVID-19 pandemic, showing observed and expected weekly deaths since January 2020 along with 95% prediction intervals. The difference between the empirical and parametric prediction intervals is especially notable during winter, when excess deaths seem far more unusual under negative binominal intervals than under the seasonally widened empirical intervals. The prediction intervals derived from the raw quantiles of the error distribution are not as smooth as the skew-normal modeled errors. Figure 6 shows the empirical skew-normal prediction intervals along with observed and forecast weekly deaths over the application data series for all 23 countries.

### Cross-country evaluation of forecast calibration

Table 1 shows calibration metrics for the three different prediction interval types as calculated from the validation series. We report the coverage and the mean interval score aggregated across all 23 countries, annually and by season, for a nominal coverage of 95%.

All three types of prediction intervals tend to be too narrow, although close to the nominal coverage. On an annual basis, the negative-binomial $95\%$ prediction intervals (PIs) perform best with an actual coverage of 93%, closely followed by the empirical skew-normal PIs with 91%. However, looking at the performance over different seasons, the empirical skew-normal PIs achieve better actual coverage in winter and autumn than the parametric PIs, as the latter are too wide in autumn (99% actual coverage) and too narrow in winter (85% actual coverage). These peculiarities are hidden when only the calibration for the whole year is considered.

These findings are also supported by the mean interval scores. The empirical skew-normal PIs are better calibrated than the negative-binomial PIs overall, and in all seasons except summer. This indicates an overall better probabilistic calibration of the empirical skew-normal PIs compared to the conventional negative-binomial PIs.

The prediction intervals derived from the raw quantiles of the forecast error distribution perform comparatively poorly which may be due to over-fitting. Thus, further results in the paper will be shown without the inclusion of the raw-quantile method.

**Table 1:** Calibration metrics for nominal 95% prediction intervals by season and type of interval. The metrics have been calculated on the test periods of the validation data (2016–2018), i.e. on data not seen either during training or calibration, and aggregated over 23 countries

	Coverage				
	Annual	Dec–Feb	Mar-May	Jun–Aug	Sep–Nov
Negative Bin. PIs	**0.93 (1)**	0.85 (3)	**0.91 (1)**	**0.95 (1)**	0.99 (3)
Empirical SN PIs	0.91 (2)	**0.89 (1)**	0.89 (2)	0.91 (2)	**0.94 (1)**
Empirical RQ PIs	0.86 (3)	0.86 (2)	0.82 (3)	0.86 (3)	0.91 (2)
	Mean interval score				
Negative Bin. PIs	0.365 (2)	0.553 (3)	0.376 (2)	**0.287 (1)**	0.248 (2)
Empirical SN PIs	**0.346 (1)**	**0.467 (1)**	**0.369 (1)**	0.310 (2)	**0.241 (1)**
Empirical RQ PIs	0.398 (3)	0.534 (2)	0.437 (3)	0.363 (3)	0.260 (3)

Bold font highlights best/same performance among types of prediction intervals. Data Source: Short-term Mortality Fluctuations (STMF) data series, own calculations

### Probability of 10% excess deaths under pre-pandemic mortality

**Fig. 4:**
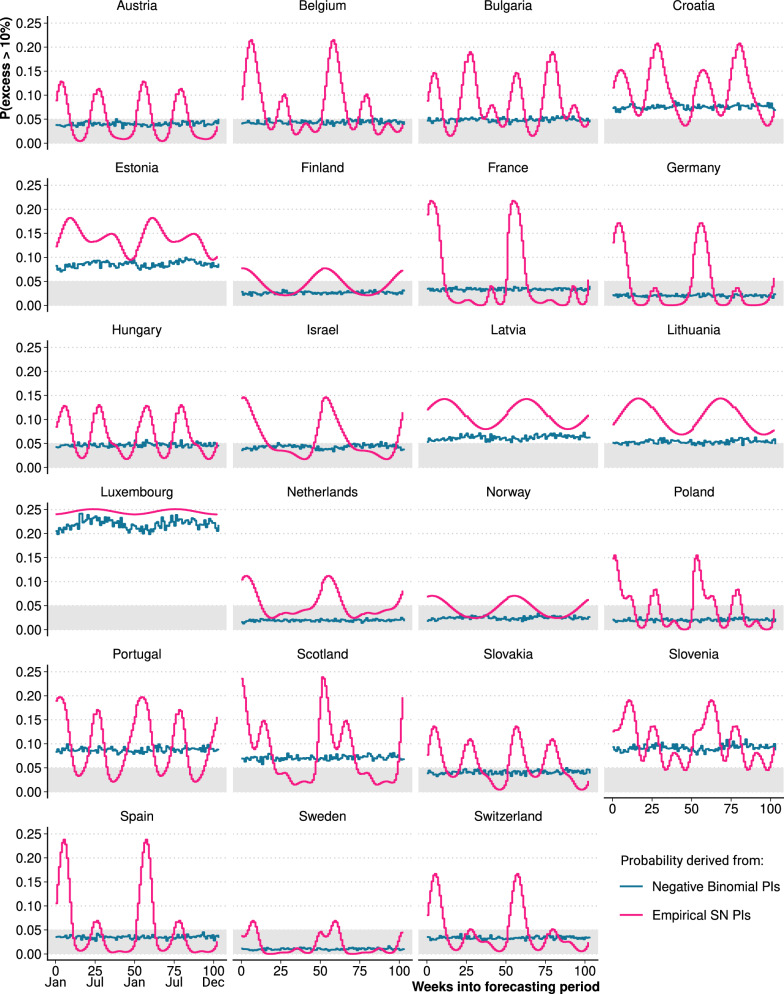
Weekly probability for 10% excess deaths given pre-pandemic mortality derived from empirical prediction intervals and negative-binomial prediction intervals for 23 European countries

**Fig. 5:**
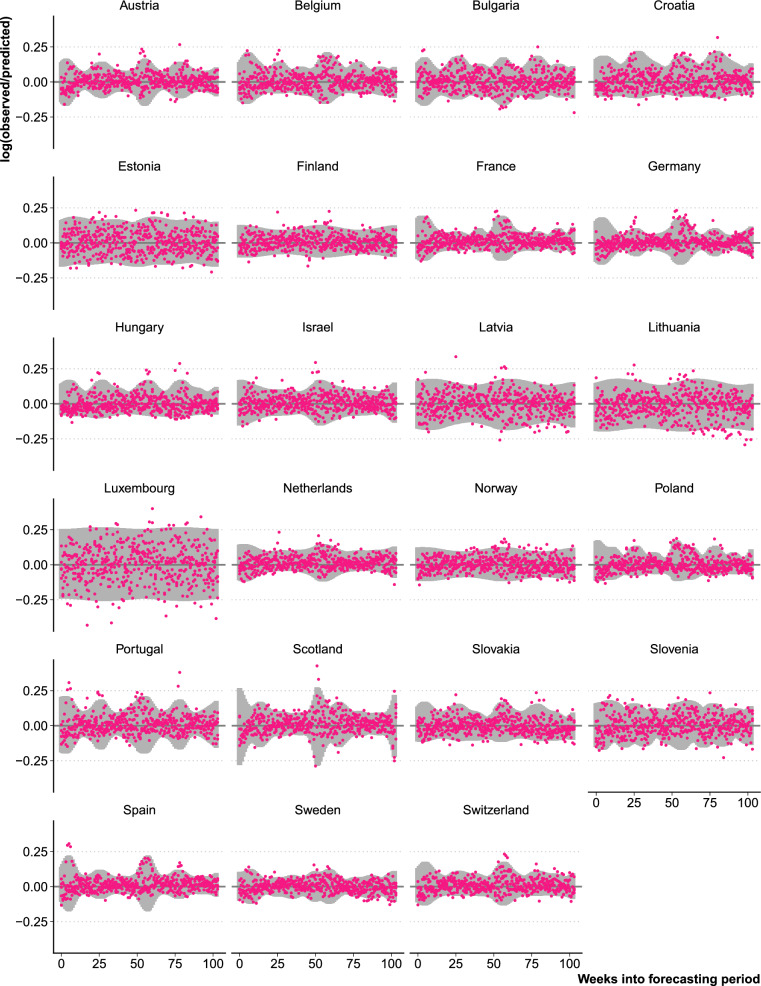
Distribution of the forecast error for weekly death counts as estimated from the calibration data series (test periods from 2006 to 2016) across 23 European countries

**Fig. 6:**
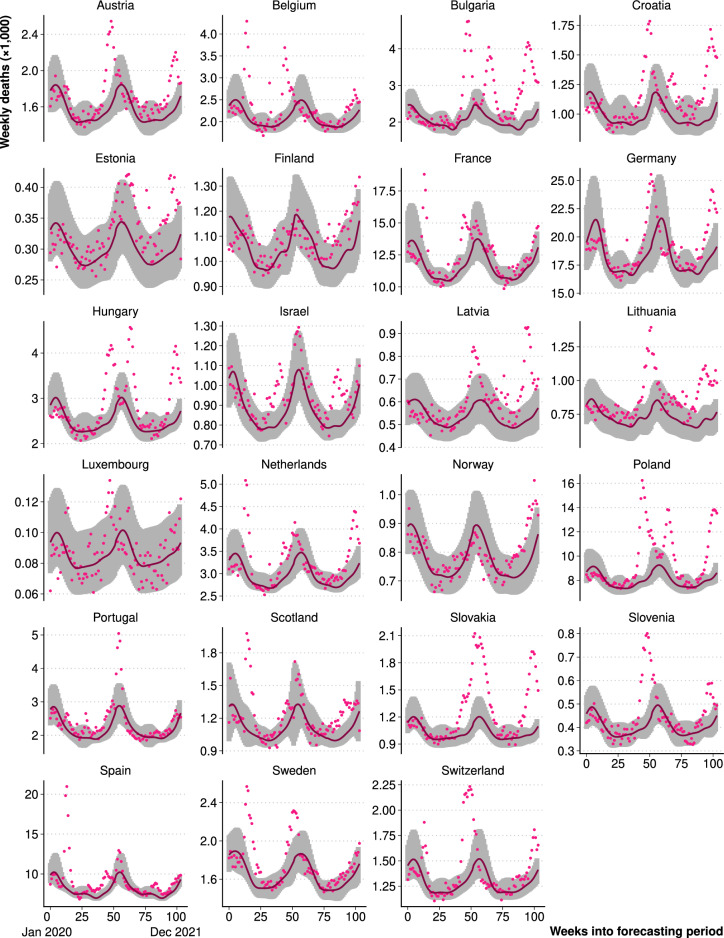
Empirical prediction intervals (95%) applied to the COVID-19 application data series (test periods from 2020 to 2021) across 23 European countries

The choice of prediction interval is crucial for evaluating the significance of a given excess death estimate. How unusual is a finding of 10% weekly excess deaths in a given country and season given non-pandemic mortality conditions? Fig. 4 answers this question for each of the 23 chosen countries under two different prediction intervals: the seasonally adjusted empirical intervals based on past forecast errors and the commonly used negative-binomial intervals with time-constant overdispersion. Under the hypothesis that past, non-pandemic mortality trends continue, the graph shows the probability (p-value) for excess deaths in a given week to exceed 10%.

If a standard negative-binomial GAM was chosen to derive the prediction intervals, a researcher would hold the belief that exceeding 10% excess death would be just as likely in spring as in winter (Fig. 4 blue line). The empirical prediction intervals (pink line) show that this is not the case and that the likelihood of exceeding the 10% threshold varies by season. A case in point is France, with a peak 20% probability of seeing at least 10% excess deaths for a winter week and a p-value approaching zero during spring. In all countries, there are periods when the p-value exceeds the level of 0.05, yet, there are differences in the seasonal patterns. For most of the countries, the probability of observing 10% excess or more is elevated during the winter and also for the summer weeks (e.g. Austria, Hungary, Portugal, Belgium, Poland, Spain). In contrast, in Norway, Sweden and Finland, we only see a winter and no summer elevation of the p-value. For Croatia and Bulgaria, the p-value is higher in summer than in winter. Therefore, across all of the 23 countries, we find periods when 10% weekly excess deaths are not unusual under normal mortality conditions. This is especially true for smaller populations (e.g. Scotland, Latvia, Luxembourg) where sampling fluctuations in death counts regularly produce excess death estimates above 10% even under normal mortality conditions. In a complementary finding, for Austria, France, Germany, Poland, Spain, and Sweden, the empirical prediction intervals predict a 10% excess p-value close to zero for parts of spring and summer, indicating that 10% excess deaths are not unusual in winter, but unusual in spring or summer.

**Fig. 7:**
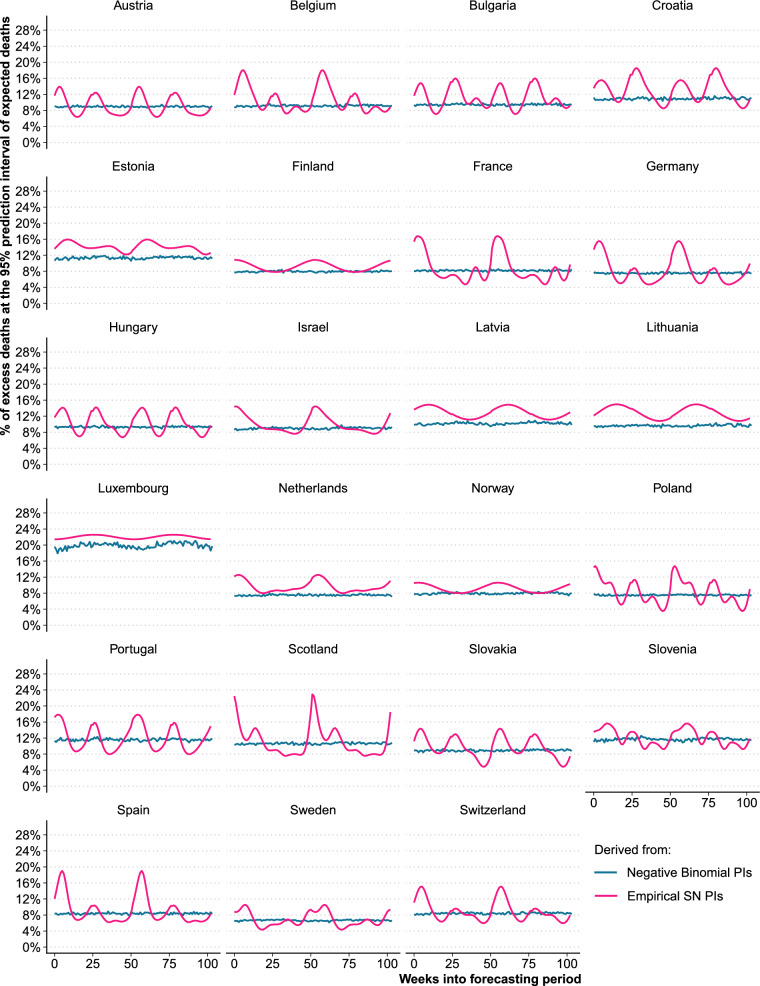
Percentage of excess deaths at the 95% prediction interval of expected deaths derived from the calibration data series (test periods from 2006 to 2016) for 23 European Countries

The question about how unusual a given level of weekly excess deaths is, can be inverted: Under pre-pandemic mortality, what level of weekly excess deaths is needed so that they fall outside e.g. a 95% prediction interval and thus be "significant"? To answer this question, we evaluated the expected death distribution at the 95% prediction interval and derived the implied excess deathsby country(Fig. 7 in the Appendix). If negative-binomial GAM prediction intervals are applied, for most countries, a level of 8–12% of excess deaths would be needed so that they fall outside of the 95% prediction interval. This level of excess deaths remains constant throughout the year. However, the use of the empirical prediction intervals reveals the seasonality of the uncertainty and that a level of excess deaths of up to 16% (and even more for smaller regions such as Scotland and Luxembourg) is needed to keep the excess deaths from being hidden by the uncertainty in the expected deaths forecasts.

## Discussion

Forecasts of weekly deaths have grown in importance since the onset of the COVID-19 pandemic and the need for estimating excess deaths. These forecasts of expected deaths have an error that is probabilistically expressed as a prediction interval. Usually, these prediction intervals are model-based parametric derivations. However, they tend to be too narrow, giving a too optimistic outlook on the precision with which one can estimate excess deaths during an ongoing pandemic. In this paper, we propose the use of empirical prediction intervals for the study of weekly expected and excess deaths and show the superior calibration of these intervals compared to parametric intervals. Further, we demonstrate the application of empirical prediction intervals to the COVID-19 pandemic, answering the question whether a 10% increase in excess deaths can be detected or whether it disappears into the uncertainty of the expected deaths forecasts.

Empirical prediction intervals are based on modeling the distribution of the forecast errors from past out-of-sample forecast errors. We use a skew-normal distribution with a time varying seasonality parameter to model the forecast errors for weekly deaths, accounting for the positive skewness and seasonality of errors. For the validation analysis, we compare the empirical prediction intervals with parametric prediction intervals obtained from a negative-binomial GAM using two measures of calibration: the coverage and the mean interval score. We obtain weekly mortality data from the Short-Term Mortality Database (STMF, [20]) for 23 countries. We showcase the calculation of empirical prediction intervals for these countries, perform the validation analyses, and apply the different intervals to excess deaths in the COVID-19 pandemic.

We showed that our model for the empirical prediction intervals is able to capture the seasonality in weekly deaths and the positive skewness due to the varying interval width over the year. In contrast, the negative-binomial prediction intervals have a constant width throughout the year, resulting in excessively wide intervals in fall and excessively narrow intervals in winter. Further, our validation analysis demonstrated that the empirical prediction intervals are better calibrated than the negative-binomial prediction intervals. This is reflected in the mean interval score that is, on average, better for the empirical prediction intervals. Both types of intervals exhibit good coverage that is, on average, slightly short of the nominal 95%.

Regarding the application question, using negative-binomial prediction intervals would give the result that a 10% increase in excess deaths would be detectable in fall of 2022. However, the empirical prediction intervals show that this is not the case during the whole year. Regarding the example of Germany, in winter a single week with 10% increase in excess deaths would disappear into the uncertainty of the forecasts of expected deaths. We found similar results for the other countries in our sample: there are periods throughout the year in which a 10% increase in weekly excess deaths can not be reliably predicted for every country. These periods differ between the countries.

The difference in forecast error seasonality between the countries may be connected to differences in the prevalent climate. Future research could further investigate this relationship between the climate of a country of interest and the seasonal patterns of uncertainty in mortality forecasting. Both the general effects of the climate and its variability from year to year are likely to shape the uncertainty in the expected deaths forecasts. A region’s climate influences population health outcomes, for example via heat-related stress. However, heatwaves are difficult to predict and usually not included in mortality forecast models, contributing to increased uncertainty in mortality forecasts for the summer months. In colder or more moderate climates, the uncertainty stems from the unpredictability of the flu season severity, which can vary significantly from year to year, increasing uncertainty during the winter months. Regarding the study of excess deaths due to COVID-19, our results highlight the importance of well calibrated prediction intervals that also address the naturally occurring seasonal uncertainty that comes with mortality forecasting.

Weeks are the common time frame for tracking the mortality during the Covid-19 pandemic, because, unlike days, they are less sensitive to reporting delays that occur on weekends and holidays, while maintaining the timeliness of the mortality monitoring. If the time frame of the analyzed death counts were changed from one week to one month or even one year, the error distribution around the expected deaths would change as well.. Determining the exact level of excess deaths needed in order to be detectable for longer time periods, is a topic for future research.

The application of empirical prediction intervals to any measure, in our case excess deaths, is based on the underlying assumption that the forecasting errors observed in the past resemble the forecasting errors in the future. One might ask whether this assumption is valid. However, as Alho et al. [33, p.42] argue, “[...] if one does not believe that they will be, it is necessary to provide arguments as to why the future is expected to be different from the past”. The counterfactual assumption that future forecasting errors will be fundamentally different from past errors is the more complicated one. Our assumption is the more conservative option, which is necessary to allow us to use empirical prediction intervals as a measure of forecast uncertainty.

Our findings are broadly generalizable in the sense that the proposed methodology is easily implemented as long as there are sufficiently long data series to perform the data splitting into calibration, validation, and application data sets. Further, we are confident that the empirical prediction intervals will outperform other types of prediction intervals for the analysis of excess deaths due to their ability to capture the seasonality in the uncertainty of the expected deaths forecasts, regardless of the country under study. However, due to the different seasonal patterns found for different countries, our findings regarding the likelihood of detecting excess deaths in a given week of the year are specific to the countries of our analysis.

Regarding applications of the proposed methodology, a real-time implementation of empirical prediction intervals for monitoring excess deaths would offer a chance to judge the “unusualness” of registered death counts. On the one hand, this would help to monitor excess deaths during a pandemic, compared to pre-pandemic mortality, and would help researchers, decision makers, and the public to assess the severity of the pandemic and to act accordingly. Empirical prediction intervals can be used, especially later in a pandemic, when counterfactual analyses become less sensible because too much time has passed. On the other hand, a real-time implementation could serve as an early indicator of public health crises due to pandemics or other environmental causes. Further, the methodology of empirical prediction intervals can increase understanding when communicating research results on excess deaths to the public. This is due to the intuitive notion that future forecast errors reflect past errors. Obviously, data on an ongoing pandemic is of great public interest. Therefore, it is important that researchers communicate their findings in a way that is easily understood not only by other researchers, but also by the public. In the case of excess deaths and the uncertainty surrounding them, we would recommend the use of confidence bands to visually convey uncertainty, rather than relying on *p*-values, for example. Further, the comparison with past observed fluctuations in death counts could help to contextualize and assess the severeness of current excess deaths.

Empirical prediction intervals are generally applicable. Not only can they capture different patterns of seasonality in deaths (e.g. high winter uncertainty and low summer uncertainty and vice versa). They can be applied to all models of excess deaths and other methods of forecasting, e.g. forecasts using expert opinions, as they only derive from observable data and not from unobservable model parameters. As long as the forecasts can be validated over a long time, empirical prediction intervals can be used. Therefore, they are not limited to mortality forecasting, either. Using adapted specifications to model the forecast error, they can be applied to e.g. fertility forecasts. Thus, research concerned with demographic forecasting in general can profit from the use of empirical prediction intervals. We invite researchers to use empirical prediction intervals for their forecasting problems and showcase the possibilities of their application.

## Data Availability

The research presented in this article is fully reproducible. We have made our codebase available to the scientific community. You can access the complete codebase, including scripts and documentation, at the following link: https://github.com/jschoeley/epunc. In addition, the data used for our analyses is sourced from openly accessible and publicly available datasets. Researchers interested in reproducing our work or conducting further investigations can freely download the data from the following link: https://www.mortality.org/Data/STMF. The availability of open-access data ensures transparency and promotes collaboration within the scientific community.

## Appendix

**Table 2:** Structure of data used for analyses

Data series	Category	Start date of training period	Start date of testing period	End date of testing period
1	Calibration	22.01.2001	23.01.2006	14.01.2008
2	Calibration	20.01.2003	21.01.2008	11.01.2010
3	Calibration	17.01.2005	18.01.2010	09.01.2012
4	Calibration	15.01.2007	16.01.2012	06.01.2014
5	Calibration	12.01.2009	13.01.2014	04.01.2016
6	Validation	10.01.2011	11.01.2016	01.01.2018
7	Validation	07.01.2013	08.01.2018	30.12.2019
8	Application	05.01.2015	06.01.2020	17.12.2021
