# Correction: Empirical prediction intervals applied to short term mortality forecasts and excess deaths

**DOI:** 10.1186/s12963-025-00362-4

**Published:** 2025-02-04

**Authors:** Ricarda Duerst, Jonas Schöley

**Affiliations:** 1 Max Planck Institute for Demographic Research, Konrad-Zuse-Straße 1, 18057 Rostock, Germany; 2 University of Helsinki, Fabianinkatu 33, Helsinki, 00014 Finland

**Correction: Population Health Metrics (2024) 22:34**

10.1186/s12963-024-00355-9

The original publication of this article contained an error in Eq. 5. The incorrect and correct equation are shown in this correction article, the original article has been updated.

Incorrect

$$\begin{aligned} \sigma _{h} = \exp \left(\alpha_{\sigma }+\beta _{\sigma}h+s_{\sigma} (w[h]) \right) \end{aligned}$$

Correct

$$\begin{aligned} \sigma _{h} = \exp \left( \alpha _{\sigma } + s_\sigma (w[h]) \right) \end{aligned}$$

